# Supplementary material for: Strong electronic interaction and multiple quantum Hall ferromagnetic phases in trilayer graphene
Source: Nat Commun. 2017 Feb 20;8:14518. doi: 10.1038/ncomms14518 (PMC5321728; doi:10.1038/ncomms14518)
Supplement: Supplementary Information — Supplementary Figures, Supplementary Tables, Supplementary Notes and Supplementary References [file ncomms14518-s1.pdf]

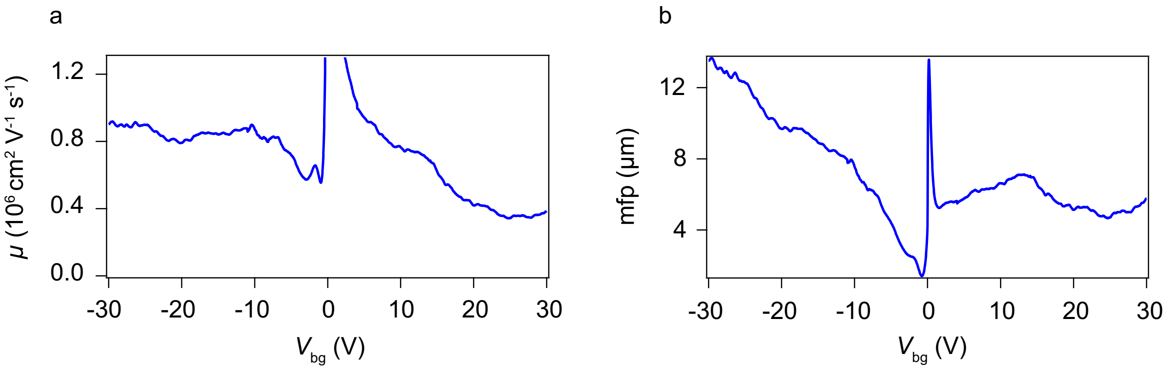

Supplementary Figure 1: Mobility and mean-free path. a) Drude mobility calculated from four-terminal resistance of the device. b) Calculated mean free path.

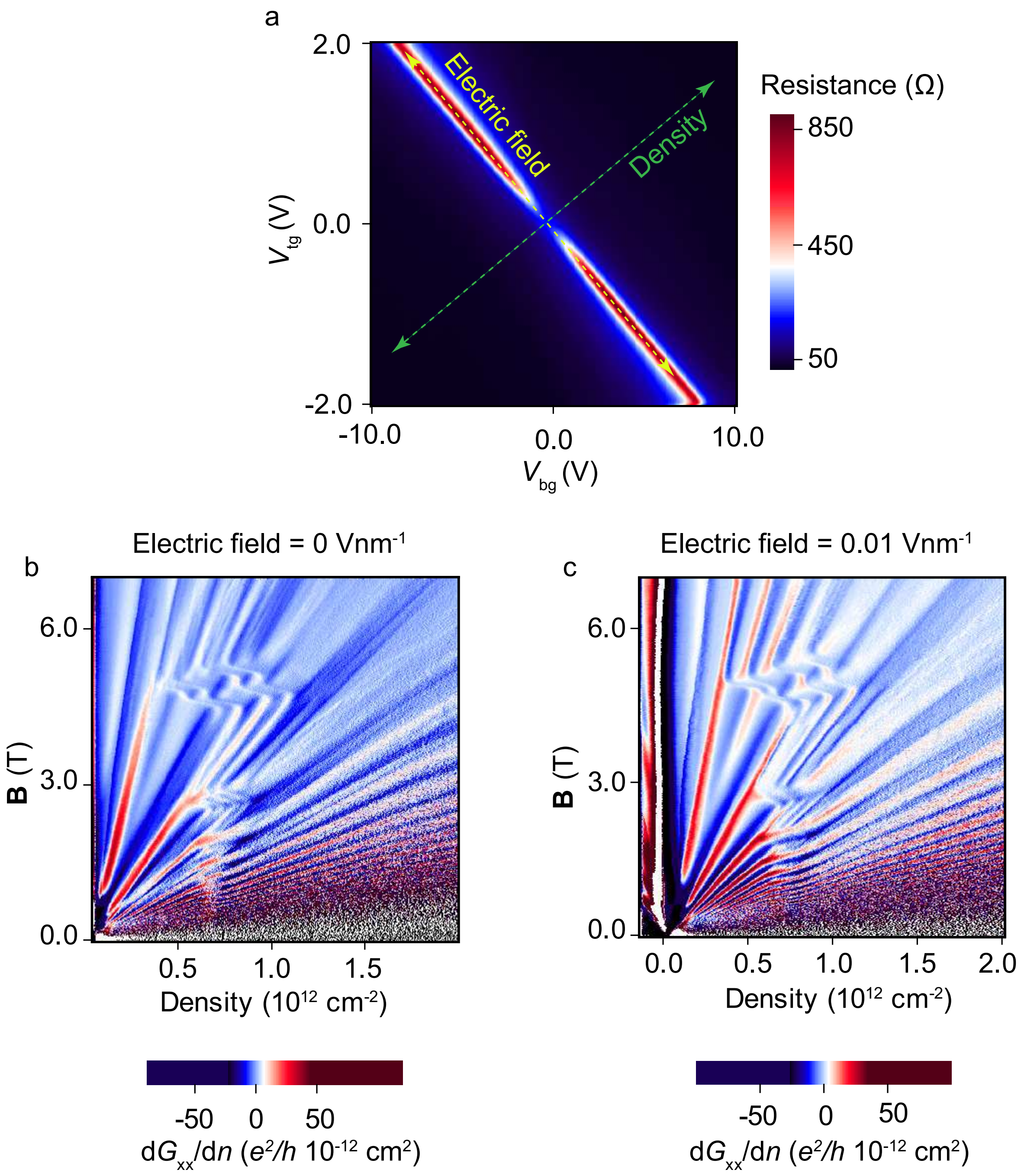

Supplementary Figure 2: Transport measurement data from dual gate device. a) Four-probe resistance as a function of back gate and top gate at zero magnetic field. Yellow and green arrows show the direction of electric field and density respectively. b) and c) show quantum Hall measurements revealing the crossings of $N_{\rm M} = 0$ and $N_{\rm B} = 2$ LLs at zero and 0.01 Vnm^{-1} electric field respectively.

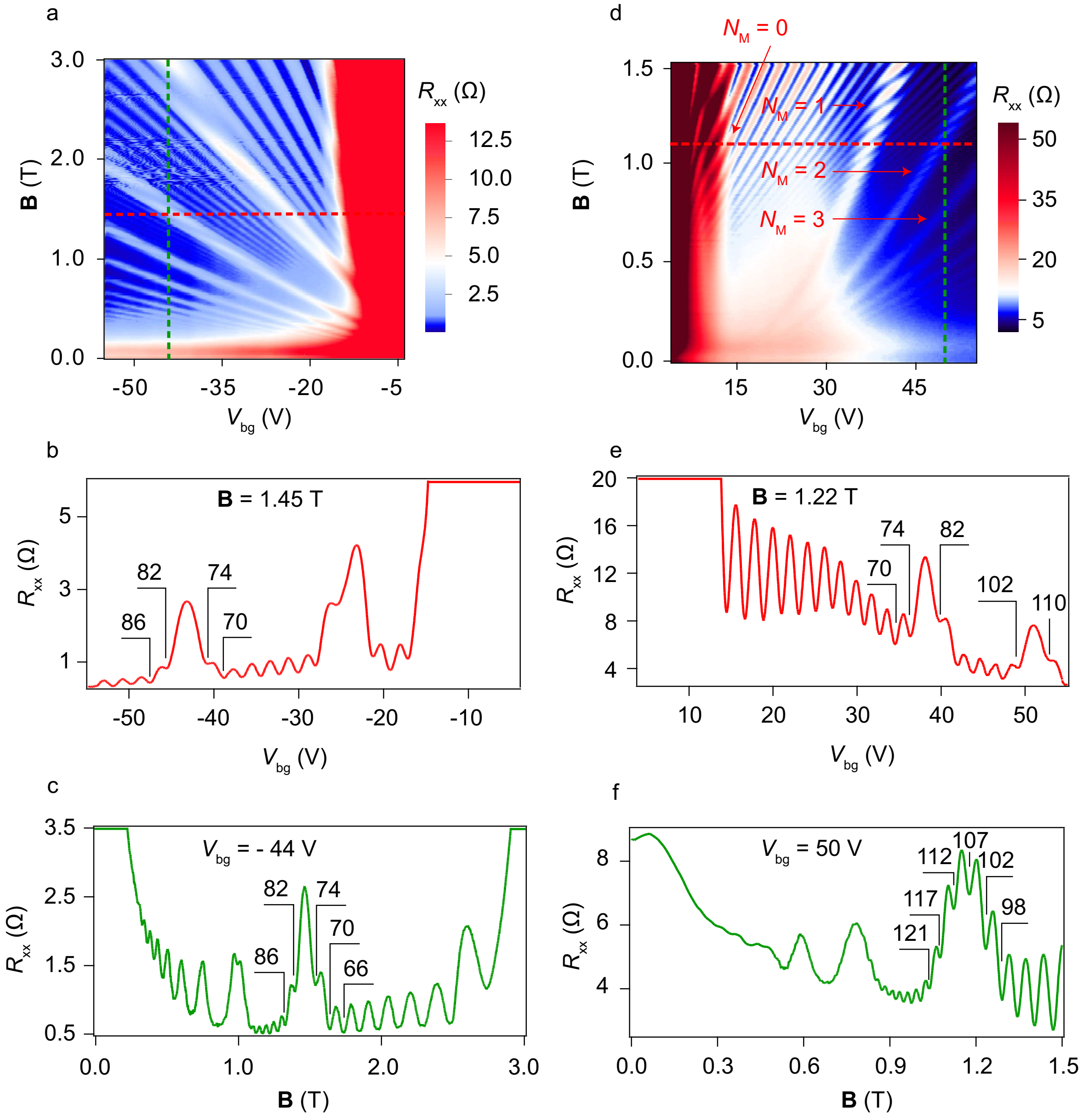

Supplementary Figure 3: Low field fan diagram. a) R_{xx} colour plot on hole side. b) Line slice of hole side data along V_{bg} axis at $\mathbf{B} = 1.45$ T. c) Line slice of hole side data along $\mathbf{B}$ axis at V_{bg} = -44 V. d) R_{xx} colour plot on electron side. e) Line slice of electron side data along V_{bg} axis at $\mathbf{B} = 1.22$ T. f) Line slice of electron side data along $\mathbf{B}$ axis at $V_{bg} = 50$ V.

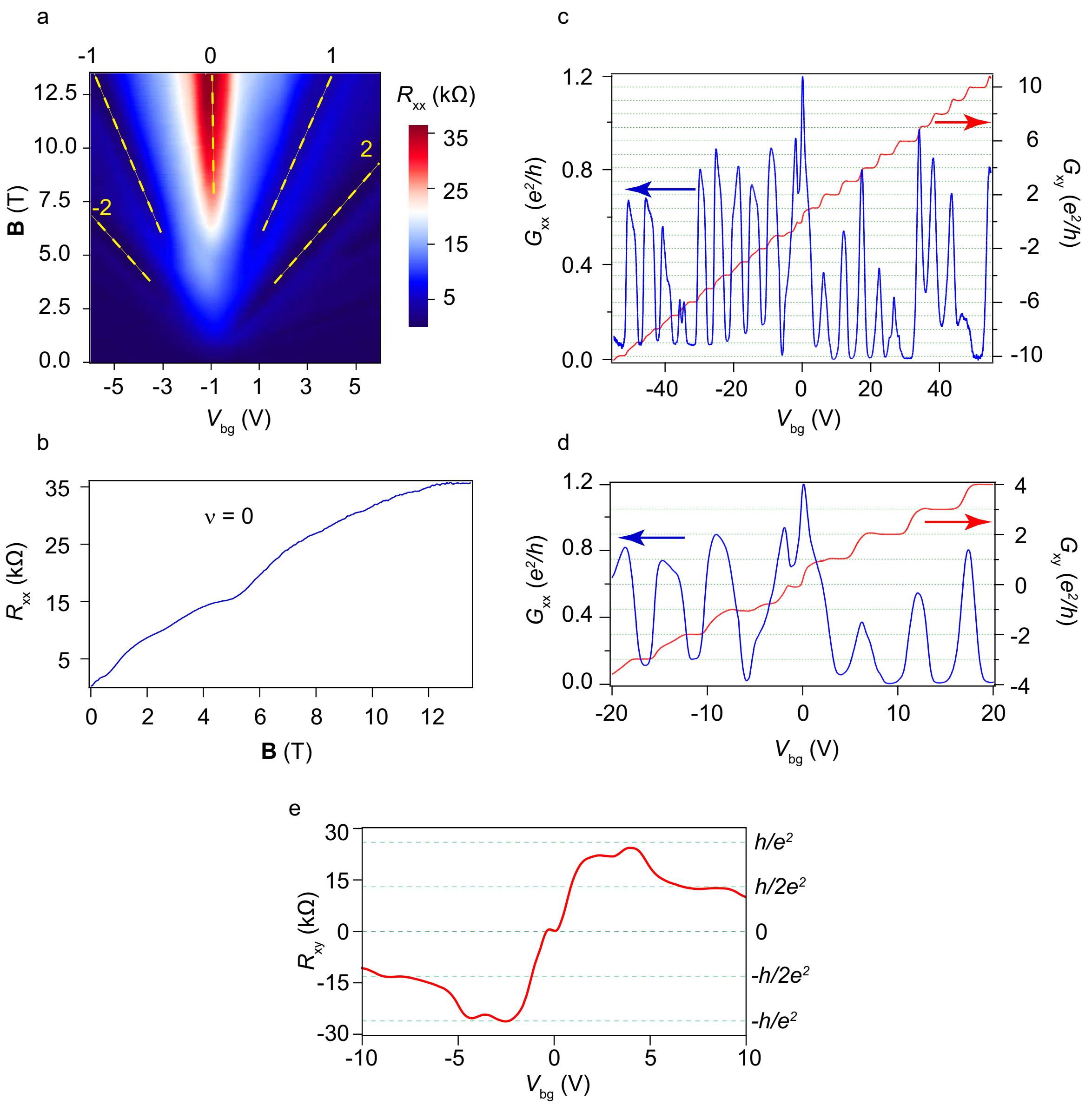

Supplementary Figure 4: Symmetry broken states. a) Colour plot of R_{xx} in the vicinity of charge neutrality point. b) Shows the evolution of R_{xx} with magnetic field at the charge neutrality point. c) G_{xx} and all G_{xy} plateaus from filling factors -10 to 10 at 13.5 T. d) Close up of Supplementary Figure 4c showing all filling factors from -3 to 4 at 13.5 T. e) R_{xy} plateaus from ν = -2 to 2 including ν = 0 at 13.5 T which shows a plateau at zero resistance.

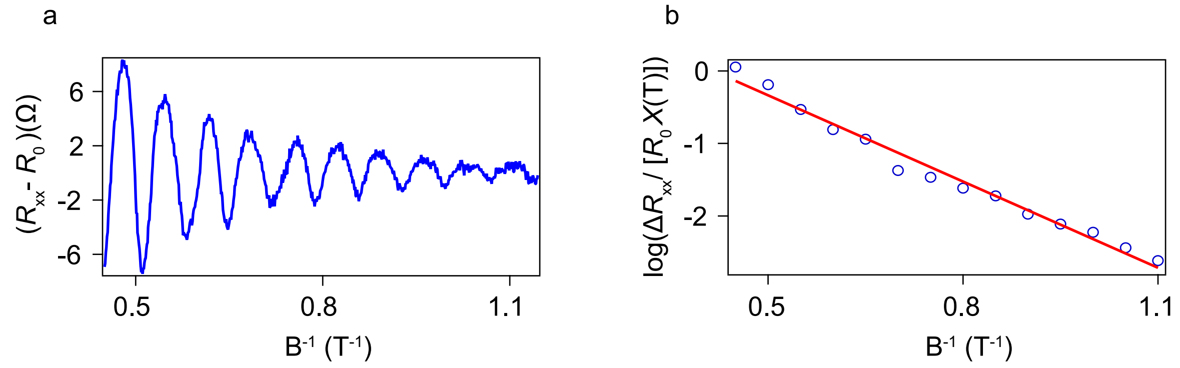

Supplementary Figure 5: Disorder strength calculation from Dingle plot on electron side at $V_{\mathrm{bg}} = 23$ V. a) SdH oscillations after subtracting the non-oscillatory part (R_0). b) Dingle plot.

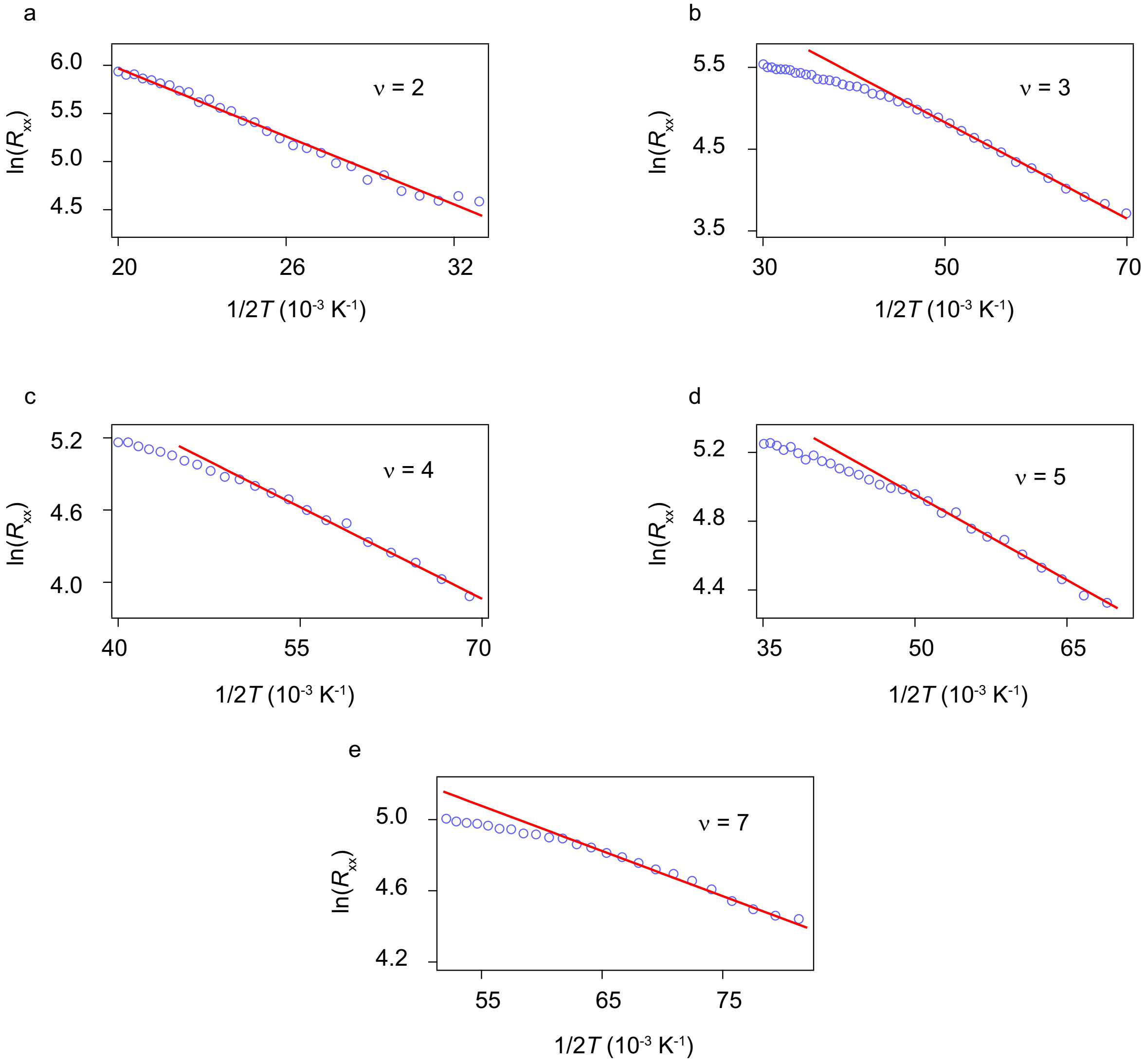

Supplementary Figure 6: Activation gap from the temperature dependence of the SdH oscillations. a) For $\nu = 2$. b) For $\nu = 3$. c) For $\nu = 4$. d) For $\nu = 5$. e) For $\nu = 7$.

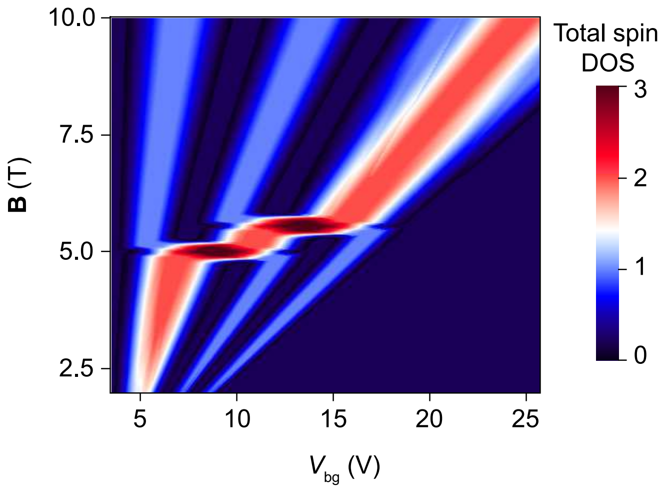

Supplementary Figure 7: Calculated total spin DOS as a function of V_{bg} and B. Monolayer-like and bilayer-like Landau level crossing regions show enhanced spin polarization.

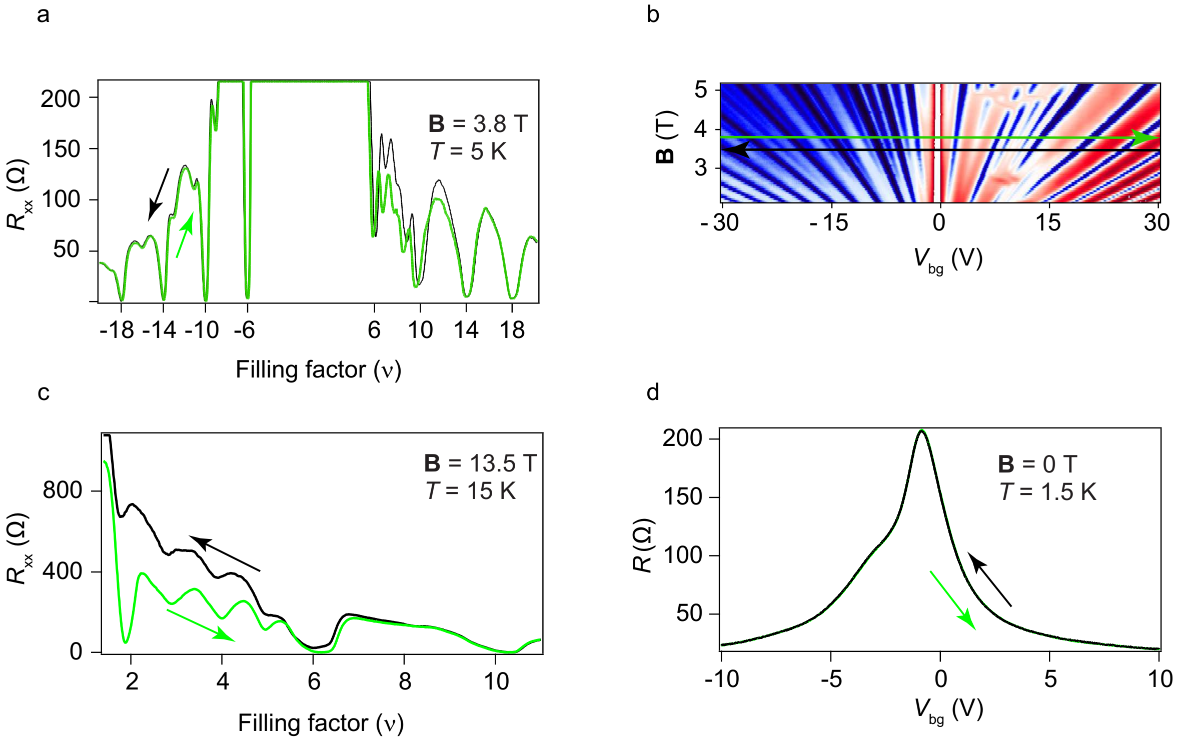

Supplementary Figure 8: Hysteresis measurements. a) Hysteresis due to $N_{\mathrm{M}} = 0$ LL at 3.8 T magnetic field. Green curve shows forward sweep and black curve shows reverse sweep in all the data. b) Parameter space of the hysteresis study at 3.8 T. c) Hysteresis study at 13.5 T and at 15 K temperature. d) V_{bg} sweep in forward and reverse direction without magnetic field, showing no hysteresis.

SUPPLEMENTARY TABLES

Supplementary Table 1: Comparison of experimental and theoretical Landau level crossing points.

LL index of monolayer-like LLs (N_{M})	LL index of bilayer-like LLs (N_{B})	Experimental magnetic field (T) at crossing point	Theoretical magnetic field (T) at crossing point
1	17	1.47	1.47
1	18	1.35	1.35
1	19	1.24	1.24
1	20	1.14	1.15
1	21	1.05	1.06
1	22	0.98	0.99
1	23	0.92	0.92
1	24	0.86	0.87
1	25	0.80	0.82
1	26	0.76	0.77

Supplementary Table 2: Extracted Landau level energy gaps from Arrhenius plots.

ν	Δ at 13.5 T (meV)
2	10.14 ± 0.02
3	5.1 ± 0.1
4	4.37 ± 0.04
5	2.8 ± 0.1
7	2.2 ± 0.1

SUPPLEMENTARY NOTE 1 : MOBILITY AND MEAN FREE PATH

We calculate Drude mobility $\mu = \frac{\sigma}{ne}$ from the four-probe resistance measurement where σ is the four-probe conductivity, μ is the mobility, n is the number density of carriers and e is the electronic charge. Hexagonal boron nitride (hBN) is used to encapsulate the ABA-TLG in this device and we find mobility is extremely large which reaches ∼500,000 $cm^2V^{-1}s^{-1}$ on electron side and even larger on hole side ∼800,000 $cm^2V^{-1}s^{-1}$.
Mean free path (mfp) of the carriers can be calculated by $mfp = \frac{\hbar\mu}{e}\sqrt{\pi n}$. Very high mobility leads to carrier mean free path in excess of 7 µm which is similar to the device size, making the conduction in ballistic regime.

SUPPLEMENTARY NOTE 2 : QUANTUM HALL MEASUREMENTS ON A DUAL GATE ABA-TRILAYER DEVICE

Supplementary Figure 2a shows the zero magnetic field data measured on a different ABA-trilayer graphene device. Supplementary Figure 2b and Figure 2c show LL crossings at two different electric fields which indicate these crossings are not very electric field sensitive at low electric field. Comparing the electric field dependence with the data presented in the main manuscript we conclude the presence of maximum stray electric field ∼0.01 Vnm^{-1} in the single gated measurements.

SUPPLEMENTARY NOTE 3 : QUANTUM HALL AT LOW MAGNETIC FIELD

It is clear from the LL energy diagram (Fig. 3a main text) that low energy regime is mostly populated by bilayer-like (BL) LLs. So, at high field (roughly $\mathbf{B} > 5$ T) no monolayer levels exist (except $N_{\mathrm{M}} = 0$) in our experimentally accessible density range -4×10^{12} cm^{-2} to 4×10^{12} cm^{-2} which corresponds to -60 V to +60 V back gate voltage. Accessing the monolayer-like (ML) LLs requires us to go to high density and very low magnetic field when the degeneracy of LLs is low so that electrons can occupy high LL index energy levels. Usually, the sample quality makes it harder to resolve LLs at such low field (∼0.5 T - 3 T), possibly it explains the reason why so far crossings of ML and BL LLs were not observed at such a low field in the fan diagram. Supplementary Figure 3a and Figure 3d show the LL fan diagram at low magnetic field on hole and electron side respectively, which shows

parabolically dispersed crossing points between different ML and BL LLs. Supplementary Figure 3b and Figure 3c show the line slices of the hole side data along V_{bg} axis at 1.45 T and along $\mathbf{B}$ axis at V_{bg} = -44 V respectively. Similarly, Supplementary Figure 3e and Figure 3f show the line slices of the electron side data along V_{bg} axis at 1.22 T and along $\mathbf{B}$ axis at $V_{\mathrm{bg}} = 50$ V respectively. All the line slices show large R_{xx} peak at the crossing points. It is noticeable that only for $\mathbf{B} > 1$ T magnetic field BL LLs start to resolve whereas the presence of ML LLs can be seen in fan diagram (via crossings) at much lesser magnetic field ∼0.25 T which is consistent with smaller cyclotron frequency of bilayer-like band compared to the monolayer-like band. It should be emphasized that there is no way to distinguish between the linear dispersing BL LLs and square root dispersing ML LLs in the fan diagram as fan diagram shows only constant filling factor lines which are always linear for a particular filling factor $\nu = \frac{nh}{Be}$ where n is the number density of carriers, given by $ne = C_{\mathrm{bg}}V_{\mathrm{bg}}$. That is why both in monolayer and bilayer graphene constant filling factor regions in fan diagram are a set of straight lines but having different slope depending on different Hall quantization. However, the crossings in the fan diagram actually help to realize the presence of two sets of differently dispersing levels: every crossing gives rise to high DOS at the crossing points and it shows up as longitudinal conductance maxima. Longitudinal conductance G_{xx} is given by $G_{\mathrm{xx}} = \frac{R_{\mathrm{xx}}}{R_{\mathrm{xx}}^2 + R_{\mathrm{xy}}^2}$, and $R_{\mathrm{xy}} \gg R_{\mathrm{xx}}$ at a Hall resistance plateau, so the formula reduces to $G_{\mathrm{xx}} \approx \frac{R_{\mathrm{xx}}}{R_{\mathrm{xy}}^2}$ other than $\nu = 0$ plateau. Hence for any quantum Hall plateau other than $\nu = 0$, conductance maxima also show up as resistance maxima. Now these crossings at low field appear at some discrete points in the fan diagram on a set of parabola as ML LLs disperse as $E_{\mathrm{M}} \sim \sqrt{B}$ and BL LLs disperse as $\mathrm{E_B} \sim \mathbf{B}$.

Experimental Landau level crossing points can be used to determine the hopping parameters. Here we have used the crossing points of monolayer-like $N_{\mathrm{M}} = 1$ Landau level (LL) with other bilayer-like Landau levels from LL index $N_{\mathrm{B}} = 17$ to $N_{\mathrm{B}} = 26$ to calculate different hopping parameters. Since, γ_0 and γ_1 are known very precisely, we vary relatively smaller hopping parameters γ_2, γ_5 and δ to match the experimentally observed magnetic field values at the crossing points. We did not include γ_3 and γ_4 for two reasons – first, there exists a very well-known approximation [1, 2] for low energy spectra which shows that an analytical solution of the non-interacting Hamiltonian is possible. Under this approximation higher order terms containing γ_3 and γ_4 are neglected. Second reason is, we did interaction calculations later using these hopping parameters. We used this approximation to calcu-

late the analytical wavefunctions of the non-interacting Hamiltonian and then used these wavefunctions to calculate the exchange energy correction self-consistently. Without the analytical wavefunctions the exchange energy calculation becomes very difficult and hence we use this approximation. Experimentally Landau level orbital index of the Landau levels is determined by the help of filling factors determined from the experimental Hall conductance (G_{xy}) plot. The experimental crossing points of different monolayer-like and bilayer-like LLs used to determine the hopping parameters and their comparison with the theoretical ones (after obtaining the final band parameters) are shown in Supplementary Table 1.

SUPPLEMENTARY NOTE 4 : $\nu = 0$ AND OTHER SYMMETRY BROKEN STATES

Supplementary Figure 4a shows the R_{xx} corresponding to the G_{xx} shown in the main text (Fig. 3c). It shows very high resistance (Supplementary Figure 4b) at charge neutrality point, the corresponding R_{xy} shows a plateau at zero resistance, indicating the occurrence of the $\nu = 0$ state. Though R_{xx} and R_{xy} are the experimentally measured quantities, G_{xx} and G_{xy} are more fundamental in theory [3] and are related as $R_{\mathrm{xx}} = \frac{G_{\mathrm{xx}}}{G_{\mathrm{xx}}^2 + G_{\mathrm{xy}}^2}$. At $\nu = 0$ state both G_{xx} and G_{xy} go to zero, hence R_{xx} starts showing very high value which increases with magnetic field. In our device, though $\nu = 0$ state starts developing from 6.5 T, it is still not fully developed till 13.5 T. Supplementary Figure 4c and Figure 4d show the observed integer quantum Hall plateaus at 13.5 T. Supplementary Figure 4e shows a plateau at zero Hall resistance indicating the formation of $\nu = 0$ state.

SUPPLEMENTARY NOTE 5 : DISORDER STRENGTH AND QUANTUM SCATTERING TIME CALCULATION FROM DINGLE PLOT

We estimate disorder strength Γ from the magnetic field dependence of the SdH oscillations which is well studied in semiconductor 2DES [4, 5] and recently in graphene [5, 6]. We calculate single particle quantum broadening (Γ) of the LLs which is related with single particle scattering time or the quantum scattering time (τ_{q}) by $\Gamma = \frac{\hbar}{2\tau_{\mathrm{q}}}$. Magnetic field dependence of SdH oscillations is given by [4, 5] $\frac{\Delta R}{R_0} = 4X(T)exp(-\pi/\omega_{\mathrm{c}}\tau_{\mathrm{q}})$ where $\omega_{\mathrm{c}} = \frac{eB}{m^*}$ is the cyclotron frequency. ΔR and R_0 are the oscillatory and non-oscillatory

part of the resistance respectively. $X(T)$ is the temperature dependent amplitude, given by $X(T) = \frac{2\pi^2 k_\mathrm{B}T/\hbar\omega_\mathrm{c}}{sinh(2\pi^2 k_\mathrm{B}T/\hbar\omega_\mathrm{c})}$.

Supplementary Figure 5a shows the low temperature (1.5 K) SdH oscillations at $V_\mathrm{bg} = 23$ V after subtracting the non-oscillatory background resistance. Supplementary Figure 5b shows a plot of the logarithm of the ratio of the oscillatory part (ΔR) and non-oscillatory part (R_0), normalised by the temperature dependent amplitude ($X(T)$) with inverse of magnetic field. This is known as Dingle plot. The red line is the straight line fit where the slope is given by $-\pi m^*/e\tau_\mathrm{q}$. We fit the SdH oscillation data in the magnetic field range where only BL LLs are present and hence we use the BL electron effective mass which is given by $m^* = \frac{\gamma_1}{\sqrt{2}v^2}$, γ_1 hopping parameter is defined in the main text, $v \approx 10^6\ ms^{-1}$ is the Fermi velocity in graphene.

On electron side, we calculate quantum scattering time from the slope $\tau_\mathrm{q} \approx 218$ fs which yields $\Gamma \sim$1.5 meV. Transport scattering time which is given by $\tau_\mathrm{t} = \frac{m^*\mu}{e}$ turns out to be ≈ 10755 fs using mobility $\mu = 3.9 \times 10^5$ cm^{2}V^{-1}s^{-1} at the same density. The large ratio of transport scattering time and quantum scattering time in our device ($\frac{\tau_\mathrm{t}}{\tau_\mathrm{q}} \approx 49$) is consistent with high mobility 2DES [4]. Transport scattering time is not very sensitive to small angle scattering, it is the large angle scattering of the carriers which decreases the mobility and hence reduces the transport scattering time. Large $\tau_\mathrm{t}/\tau_\mathrm{q}$ indicates that small angle long range Coulomb scattering is the dominant scattering mechanism in our device [4, 5, 7].

SUPPLEMENTARY NOTE 6 : ACTIVATION GAP FOR SYMMETRY BROKEN STATES $\nu = 2, 3, 4, 5, 7$

Supplementary Figure 6 shows the activation gap determination from the Arrhenius plots calculated for $\nu = 2, 3, 4, 5, 7$. The error is dominated by the uncertainty to find the linear region of the Arrhenius plot which is much larger than the standard deviation of fitting due to scattered data. Extracted Landau level energy gaps from Arrhenius plots for different filling factors are shown in Supplementary Table 2.

SUPPLEMENTARY NOTE 7 : DETAILS OF THEORETICAL CALCULATIONS

The Hamiltonian of an ABA trilayer graphene which is symmetric under the exchange of the two outermost layers (i.e. no electric field between the layers), decouples into a monolayer-like block consisting of co-ordinates antisymmetric under exchange of the outer layers (i.e. $\left(\frac{A_1-A_3}{\sqrt{2}}, \frac{B_1-B_3}{\sqrt{2}}\right)$) and a bilayer-like block with the low energy co-ordinates $\left(\frac{A_1+A_3}{\sqrt{2}}, B_2\right)$ [8, 9]. Here $A(B)$ denotes the sublattice and $1, 2, 3$ denote the layer indices. In a perpendicular magnetic field, the energy of the monolayer-like and bilayer-like LLs are given by

$$\epsilon^{\mathrm{m}}_{N\tau} = a + \tau\sqrt{b^2 + \frac{2\hbar^2 v^2}{l_B^2}N} \quad \text{and} \tag{1}$$

$$\begin{aligned}\epsilon^{\mathrm{b}}_{N\tau} &= c + \frac{\hbar^2 v^2}{2\gamma_1^2 l_B^2}(2Nd - d - \tau f) \\ &+ \sqrt{\left[\left(c + \frac{\hbar^2 v^2}{2\gamma_1^2 l_B^2}(2Nf - \tau d - f)\right)^2 + \left(\frac{\hbar^2}{m l_B^2}\sqrt{N(N-1)}\right)^2\right]}\end{aligned} \tag{2}$$

where $a = \left(\frac{\delta}{2} - \frac{\gamma_2}{4} - \frac{\gamma_5}{4}\right)$, $b = \left(-\frac{\delta}{2} - \frac{\gamma_2}{4} + \frac{\gamma_5}{4}\right)$, $c = \frac{\gamma_2}{4}$, $d = (\delta + \frac{\gamma_5}{4})$, and $f = -\frac{\gamma_5}{4}$, with $l_B = \sqrt{\frac{\hbar}{eB}}$, $\frac{1}{m} = \frac{\sqrt{2}v^2}{\gamma_1}$. Here v is the graphene Fermi velocity and γ_i, δ are the band parameters described in the text. For the $N = 0$ monolayer-like LL, the energies are given by $\epsilon^{\mathrm{m}}_{0\pm} = a \pm b$, leading to a valley splitting of these levels $\sim 2b = 2meV$ with the chosen band parameters. In terms of the non-relativistic landau level orbitals $|N\rangle$ at a given guiding center position, the eigenfunctions for the $N = 0$ LL are given by [8]

$$\Phi^{\mathrm{m}}_{0+} = \begin{pmatrix} 0 \\ |0\rangle \end{pmatrix} \quad \text{and} \quad \Phi^{\mathrm{m}}_{0-} = \begin{pmatrix} |0\rangle \\ 0 \end{pmatrix} \tag{3}$$

and the eigenfunctions for the bilayer-like LLs are given by

$$\Phi^{\mathrm{b}}_{N+} = \begin{pmatrix} u_{N+}|N-2\rangle \\ v_{N+}|N\rangle \end{pmatrix} \quad \text{and} \quad \Phi^{\mathrm{b}}_{N-} = \begin{pmatrix} u_{N-}|N\rangle \\ v_{N-}|N-2\rangle \end{pmatrix} \tag{4}$$

where, $u_{N\tau}$ and $v_{N\tau}$ are given by $u^2_{N\tau} = \frac{1}{2}\left(1 + \frac{c + \frac{\hbar^2 v^2}{2\gamma_1^2 l_B^2}(2Nf - \tau d - f)}{\sqrt{\left(c + \frac{\hbar^2 v^2}{2\gamma_1^2 l_B^2}(2Nf - \tau d - f)\right)^2 + \left(\frac{\hbar^2}{m l_B^2}\sqrt{N(N-1)}\right)^2}}\right)$ and $v^2_{N\tau} = 1 - u^2_{N\tau}$.

We consider the effects of disorder and electronic interactions in the following way. First we assume that the exchange correction leads to modified energy levels $E^{\mathrm{m(b)}}_{N\sigma\tau} = \epsilon^{\mathrm{m(b)}}_{N\tau} +$

$g_0\mu_B B\sigma + \Delta^{\rm m(b)}_{N\sigma\tau}$, where Δ is the exchange self-energy. We note that we use the same 2D bare Coulomb potential $V(q) = 2\pi e^2/\kappa q$ for interaction between electrons in different layers, where we use $\kappa = 4$, corresponding to the dielectric constant of hBN, since the sample has layers of hBN on either side of the TLG. This neglects variation of the potential on a scale of layer separation $\sim 3-4$ Å , and is justified when the screening length is much larger than this scale. This choice, together with the layer exchange symmetry of the trilayer, keeps the bilayer-like and monolayer-like levels decoupled even in the presence of electronic interactions. We have checked that there is no signature of spontaneous breaking of this symmetry due to interactions. In reality, keeping the z dependence of Coulomb interactions would lead to coupling of the bilayer and monolayer-like LLs, and the effects would be strongest when the LLs cross. However, precisely in this regime, the large density of states lead to strong screening and neglecting the z dependence of the Coulomb interaction is justified. We then use self-consistent Born approximation (SCBA) to construct the disorder broadened single particle Green's functions[10]

$$G^{\rm m(b)}_{N\sigma\tau}(\omega) = \frac{2}{\omega - E^{\rm m(b)}_{N\sigma\tau} + i\sqrt{\Gamma^2 - (\omega - E^{\rm m(b)}_{N\sigma\tau})^2}} \tag{5}$$

with the corresponding parabolic density of states, $\rho_{N\sigma\tau}(\omega) = \frac{1}{2\pi l_B^2}\frac{2}{\pi\Gamma}\sqrt{1-(\omega - E^{\rm m(b)}_{N\sigma\tau})^2/\Gamma^2}$, when $|\omega - E^{\rm m(b)}_{N\sigma\tau}| < \Gamma$. The disorder broadening Γ is used as an input in our theory. We use $\Gamma = 1$ meV roughly similar with experimentally estimated $\Gamma \sim 1.5$ meV at $V_{\rm bg} = 23$ V. The exchange energy is then given by

$$\Delta^{\rm m(b)}_{N\sigma\tau} = -\int \frac{d^2 q V(q)}{\varepsilon(q)} A^{\rm m(b)}_{N\tau}(q)\rho_{N\sigma\tau} \tag{6}$$

where $\rho_{N\sigma\tau}$ is the electronic density in the corresponding state and the matrix elements $A^{\rm m(b)}_{N\tau}(q) = \left|\int d^2 r \Phi^{\rm m(b)^\dagger}_{N\tau}(r).\,(e^{i\mathbf{q}.\mathbf{r}})\,\Phi^{\rm m(b)}_{N\tau}(r)\right|^2$. The dielectric function $\varepsilon(q) = 1 + V(q)\Pi(q)$, where the bare polarizability function Π is given by

$$\Pi(q) = -\frac{1}{\beta}\sum_{i\omega}\sum_{\substack{N\sigma\tau \\ \{\rm m,b\}}} G^{\rm m(b)}_{N\sigma\tau}(i\omega)G^{\rm m(b)}_{N\sigma\tau}(i\omega)A^{\rm m(b)}_{N\tau}(q). \tag{7}$$

Here we have neglected inter-LL couplings in calculating the static polarizability function. With these approximations, the density in each level is calculated self-consistently. The corresponding $V_{\rm bg}$ is computed using the capacitance of the device and the resulting DOS at

the Fermi level is plotted (Fig. 4a) as a function of magnetic field **B** and the V_{bg} to match the experimental data on G_{xx}. Supplementary Figure 7 shows the total spin polarization corresponding to Fig. 4b in the main text which shows spin polarization at the Fermi energy. As seen from the Supplementary Figure 7, total spin polarization is more at the crossing points of LLs.

SUPPLEMENTARY NOTE 8 : HYSTERESIS STUDY VARYING TEMPERATURE AND MAGNETIC FIELD

In the main text, hysteresis data is shown for high field (13.5 T) which indicates the pseudospin ordering in the QHF states. Supplementary Figure 8a shows the hysteresis in R_{xx} across the symmetry broken $N_{\mathrm{M}} = 0$ LL at low magnetic field 3.8 T. Even at such a low magnetic field $N_{\mathrm{M}} = 0$ LL is completely symmetry broken and gives rise to these QHF states. Supplementary Figure 8c shows the hysteresis at 13.5 T measured at 15 K. Beyond this temperature LLs are not resolved properly. Supplementary Figure 8d shows resistance recorded at zero magnetic field with gate sweep which shows no hysteresis. It implies that hysteresis near the QHF states comes from the pseudospin order, and not from the charge traps in SiO_2/Si^{++} substrate.

SUPPLEMENTARY REFERENCES

[1] McCann, E. & Fal'ko, V. I. Landau-level degeneracy and quantum Hall effect in a graphite bilayer. *Phys. Rev. Lett.* **96,** 086805 (2006).

[2] Koshino, M. & McCann, E. Parity and valley degeneracy in multilayer graphene. *Phys. Rev. B* **81,** 115315 (2010).

[3] Sarma, S. D. & Yang, K. The enigma of the $\nu = 0$ quantum Hall effect in graphene. *Solid State Commun.* **149,** 1502 – 1506 (2009).

[4] Coleridge, P. T. Small-angle scattering in two-dimensional electron gases. *Phys. Rev. B* **44,** 3793 (1991).

[5] Hwang, E. H. & Sarma, S. D. Single-particle relaxation time versus transport scattering time in a two-dimensional graphene layer. *Phys. Rev. B* **77,** 195412 (2008).

[6] Hong, X., Zou, K. & Zhu, J. Quantum scattering time and its implications on scattering sources in graphene. *Phys. Rev. B* **80,** 241415 (2009).

[7] Knap, W. *et al.* Spin and interaction effects in Shubnikov-de Haas oscillations and the quantum Hall effect in GaN/AlGaN heterostructures. *Journal of Physics: Condensed Matter* **16,** 3421 (2004).

[8] Serbyn, M. & Abanin, D. A. New Dirac points and multiple Landau level crossings in biased trilayer graphene. *Phys. Rev. B* **87,** 115422 (2013).

[9] Koshino, M. & McCann, E. Gate-induced interlayer asymmetry in ABA-stacked trilayer graphene. *Phys. Rev. B* **79,** 125443 (2009).

[10] Ando, T. & Uemura, Y. Theory of oscillatory g factor in an MOS inversion layer under strong magnetic fields. *J. Phys. Soc. Jpn.* **37,** 1044 – 1052 (1974).
